# Fine Mapping of the Mouse *Ath28* Locus Yields Three Atherosclerosis Modifying Sub-Regions

**DOI:** 10.3390/genes13010070

**Published:** 2021-12-28

**Authors:** Juying Han, Brian Ritchey, Emmanuel Opoku, Jonathan D. Smith

**Affiliations:** Department of Cardiovascular & Metabolic Sciences, Cleveland Clinic, Cleveland, OH 44195, USA; hanj@ccf.org (J.H.); ritcheb@outlook.com (B.R.); opokue@ccf.org (E.O.)

**Keywords:** mouse genetics, quantitative trait locus mapping, atherosclerosis

## Abstract

A mouse strain intercross between *Apoe*^−/−^ AKR/J and DBA/2J mice identified three replicated atherosclerosis quantitative trait loci (QTLs). Our objective was to fine map mouse atherosclerosis modifier genes within a genomic region known to affect lesion development in apoE-deficient (*Apoe*^−/−^) mice. We dissected the *Ath28* QTL on the distal end of chromosome 2 by breeding a panel of congenic strains and measuring aortic root lesion area in 16-week-old male and female mice fed regular laboratory diets. The parental congenic strain contained ~9.65 Mb of AKR/J DNA from chromosome 2 on the DBA/2J genetic background, which had lesions 55% and 47% smaller than female and male DBA/2J mice, respectively (*p* < 0.001). Seven additional congenic lines identified three separate regions associated with the lesion area, named *Ath28.1*, *Ath28.2*, and *Ath28.3*, where the AKR/J alleles were atherosclerosis-protective for two regions and atherosclerosis-promoting for the other region. These results were replicated in both sexes, and in combined analysis after adjusting for sex. The congenic lines did not greatly impact total and HDL cholesterol levels or body weight. Bioinformatic analyses identified all coding and non-coding genes in the *Ath28.1* sub-region, as well as strain sequence differences that may be impactful. Even within a <10 Mb region of the mouse genome, evidence supports the presence of at least three atherosclerosis modifier genes that differ between the AKR/J and DBA/2J mouse strains, supporting the polygenic nature of atherosclerosis susceptibility.

## 1. Introduction

There are several mouse models of elevated non-HDL cholesterol that drive atherosclerosis in large arteries, including apoE-deficient, LDL-receptor deficient, LDL-receptor knockdown, and apoB-transgenic [1]. Classical mouse genetics using strain intercrosses have been utilized to identify quantitative trait loci (QTLs) [2] associated with lesion severity, in order to map the position and ultimately identify atherosclerosis modifier genes in an unbiased fashion. For example, the Breslow lab using *Apoe*^−/−^ mice on the C57BL/6J and FVB/J strains mapped the atherosclerosis (Ath) *Ath11* QTL on chromosome 10, and identified *Raet1e* as the causal gene [3]. Similarly, Maeda and colleagues mapped the aortic arch atherosclerosis (Aath) *Aath4* locus to distal chromosome 2 using 129S6 and DBA/2 *Apoe*^−/−^ strains, and suggested that the *Mertk* gene, with decreased expression in DBA/2 macrophages was the causal gene at this locus [4]. We have been studying *Apoe*^−/−^ mice on the AKR/J and DBA/2J strains, as aortic root lesion areas differ by ~10-fold in these strains, with DBA/2J having the largest lesions of six *Apoe*^−/−^ strains studied, while AKR/J was one of several strains with the small lesions [5]. We performed two independent strain intercrosses and identified three reproducible atherosclerosis QTLs on chromosomes 2, 15, and 17, named *Ath28*, *Ath22*, and *Ath26*, respectively [6]. The goal of our study was to perform fine mapping for the *Ath28* QTL locus and then identify candidate genes within the fine mapped locus. In the current study, we backcrossed the F1 hybrids onto the DBA/2J background, preserving the donor *Ath28* locus from the AKR/J strain; we found that the AKR/J region confers an approximately two-fold effect on aortic root atherosclerosis in this congenic strain. Further backcrossing of the initial congenic strain was performed to identify recombinants within this interval and make additional congenic strains for fine mapping the *Ath28* locus. We mapped three sub-regions within the *Ath28* locus, two of which were atherosclerosis-protective and one of which was atherosclerosis-promoting, and we discuss candidate genes in these regions.

## 2. Materials and Methods

### 2.1. Ath28 Congenic Lines

All mouse studies were conducted in accordance with the National Institute of Health (NIH) Guide for the Care and Use of Laboratory under protocols approved by the Cleveland Clinic Animal Care and Use Committee. *Apoe*^−/−^ mice on the AKR/J and DBA/2J backgrounds were previously created by backcrossing for >20 generations while preserving the *Apoe*^−/−^ locus. These mice were interbred to generate F1 hybrid progenitors. F1 mice were backcrossed to DBA/2J *Apoe*^−/−^ mice to generate N2 mice, which were genotyped using two SNP markers (rs13476925 and rs13476938) to select N2 breeders carrying AKR/J alleles in the *Ath28* region on chromosome 2 (chr2). Backcrossing to DBA/2J *Apoe*^−/−^ mice was repeated until the N7 generation, when SNP microarray genotyping (GigaMuga or MegaMuga, Neogen Corp.) was performed to select breeders with the least AKR/J contamination outside of the *Ath28* region. Two additional backcrosses were performed to generate mice heterozygous (het) for the congenic interval DBA/2.AKR^Ch2.1-7^ (hereafter called the 1-7 line), which contained AKR/J alleles on chr2 from 171,550,518 bp to 180,092,501 bp (the last chr2 polymorphic marker on the MegaMuga array, all bp positions in mm9 build). The 1-7 line heterozygotes were brother-sister mated to generate three genotypes: AA (two AKR/J alleles in the congenic interval), AD (heterozygotes), and DD (two DBA/2 alleles). The remaining subcongenic lines were all derived by further backcrossing of the AD genotype 1-7 line to DBA/2J *Apoe*^−/−^ mice generating ~480 N2 mice that were screened for recombination using a panel of seven SNP markers (Supplementary Table S1). The subcongenic strains were named by the SNP markers defining the congenic interval endpoints, and more accurate endpoints were determined via MegaMuga SNP genotyping, with the chr2 bp positions and uncertain regions shown in Supplementary Table S2. Additional information about the 3’ end of the 1-4 line was obtained using all 10 additional AKR/J-DBA/2J polymorphic SNPs (Supplementary Table S1) in this region, that were genotyped by genomic DNA PCR and Sanger sequencing. Brother-sister matings of each subcongenic line were performed and the AA and DD genotypes were used for phenotypic analysis. The DD genotypes from each of the congenic lines were combined as the control DBA/2 genotype.

### 2.2. Phenotypic Analyses

Male and female mice were fed a regular diet (Teklad 2018) and at 16 weeks of age they were sacrificed by CO_2_ inhalation. Whole blood was collected from the retroorbital plexus into a heparinized glass capillary and transferred to a tube containing 2 µL EDTA, which was spun in a microfuge to obtain plasma. The circulatory system was perfused with 10 mL PBS and the heart was excised and fixed in 10% phosphate buffered formalin (Thermo Fisher Scientific, Waltham, MA, USA). Quantitative assessment of atherosclerosis in the aortic root was performed as previously described [7]. Lesion areas were quantified as the mean value in six 10 μm-thick sections, stained with Oil red O and hematoxylin, at 80 μm intervals using Image Pro software (Media Cybernetics, Rockville, MD, USA). Total and HDL cholesterol levels were determined as previously described [8].

### 2.3. Statistical Analysis

All data were analyzed separately for male and female mice. All data were tested for normality and if the data passed the D’Agostino–Pearson, Shapiro–Wilk, or Kolmogorov–Smirnov test, parametric one-way ANOVA was used, and if not, nonparametric Kruskal–Wallis ANOVA was performed. Multiple comparison tests were performed vs. DBA/2 controls (Dunnet’s or Dunn’s for parametric and nonparametric, respectively). For combined sex analyses, the effect of sex was adjusted for by multiplying all of the male lesion values by the mean DBA/2 female lesion area/mean DBA/2 male lesion area, thus making the mean DBA/2 male lesions = the mean DBA/2 female lesions.

### 2.4. Bioinformatic Analysis

To identify sequence differences in the *Ath28.1* region of the AKR/J and DBA/2J genomes, we converted the mm9 location (chr2:172,954,530–173,171,972) to mm10 location (chr2:173,290,029–173,346,471) in order to search on the Sanger Mouse Genomes Project Release-1505, that was shown in the mm10 assembly (https://www.sanger.ac.uk/sanger/Mouse_SnpViewer/rel-1505, accessed on 1 September 2021). SNPs, small insertion deletions (indels), and structural variations were downloaded as .csv files. Candidate protein coding genes in the interval were also searched in September 2021 on PubMed to see how many citations of the gene or protein name were recovered, with the search term “AND atherosclerosis” added. Tissue level expression of the protein coding genes was queried in humans using the GTEx Portal (https://gtexportal.org/home/, accessed on 1 September 2021). Human genome wide association study (GWAS) hits were searched in 1 September 2021 on the GWAS Catalog portal (https://www.ebi.ac.uk/gwas/), using <5 × 10^−8^ as the threshold for significance.

## 3. Results

**Figure 1:**
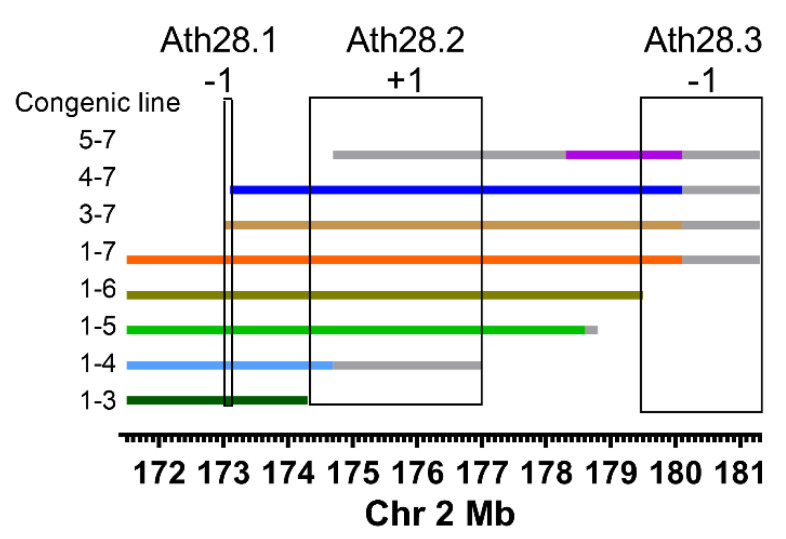
*Ath28* subcongenic strains and aortic root lesions. The map positions of the eight strains that cover this region, areas between mapped markers are shown in gray. The boxed regions show the position of three putative atherosclerosis QTLs with the −1 and +1 scores where the AKR alleles are associated with smaller and larger lesions, respectively.

**Figure 2:**
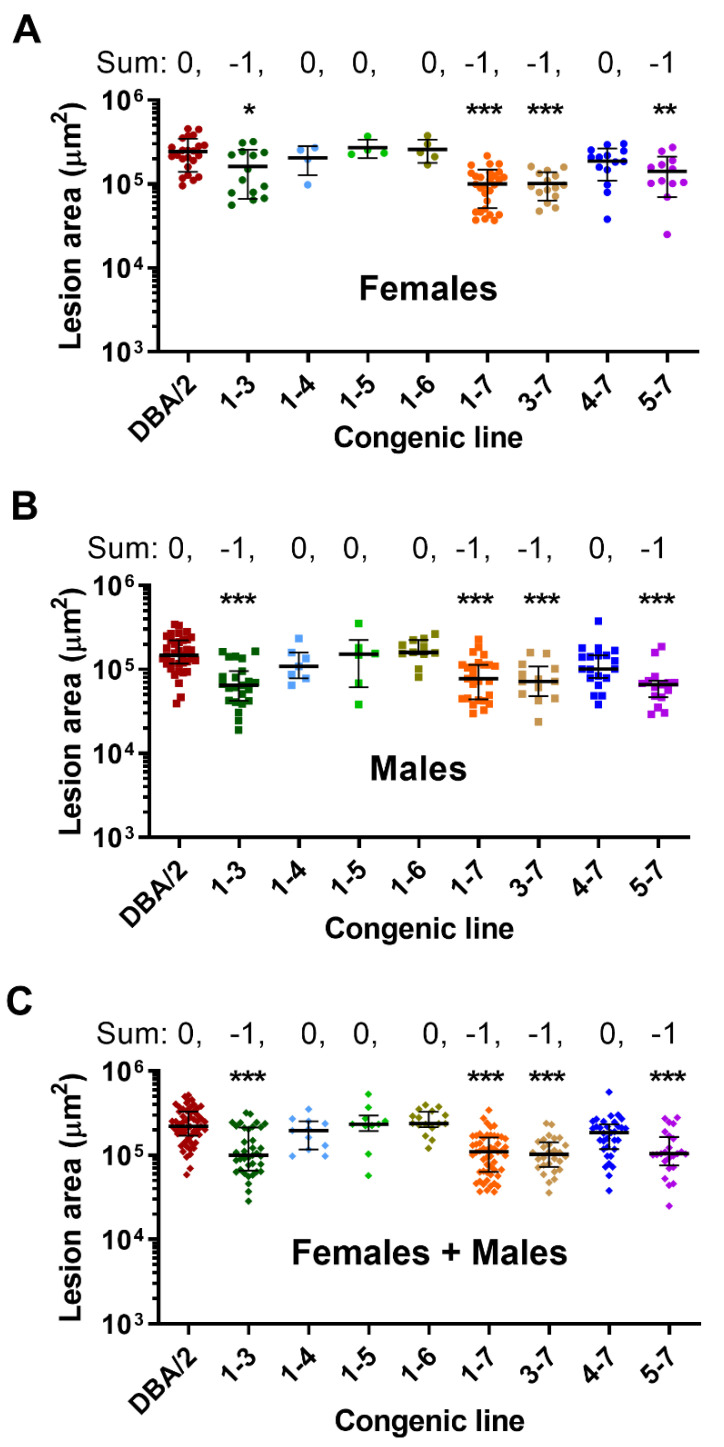
Aortic root lesion areas in female mice (**A**), male mice (**B**), and combined sexes after sex adjustment (**C**). Lesions were compared against the DBA/2J strain, in females the data were normally distributed (mean ± S.D. shown) and analyzed by one-way ANOVA and Dunnett’s multiple comparison test. Males and combined sex lesions (median ± I.Q.R shown) were analyzed by non-parametric Kruskal–Wallis test with Dunn’s multiple comparison test. *, *p* < 0.05; **, *p* < 0.01; ***, *p* < 0.001. The sum above the lesion data indicates the lesion change expected based on inclusion of the *Ath28.1* (−1, the AKR/J allele decreases lesion area), *Ath28.2* (+1, the AKR/J allele increases lesion area), and *Ath28.3* regions (−1).

**Figure 3:**
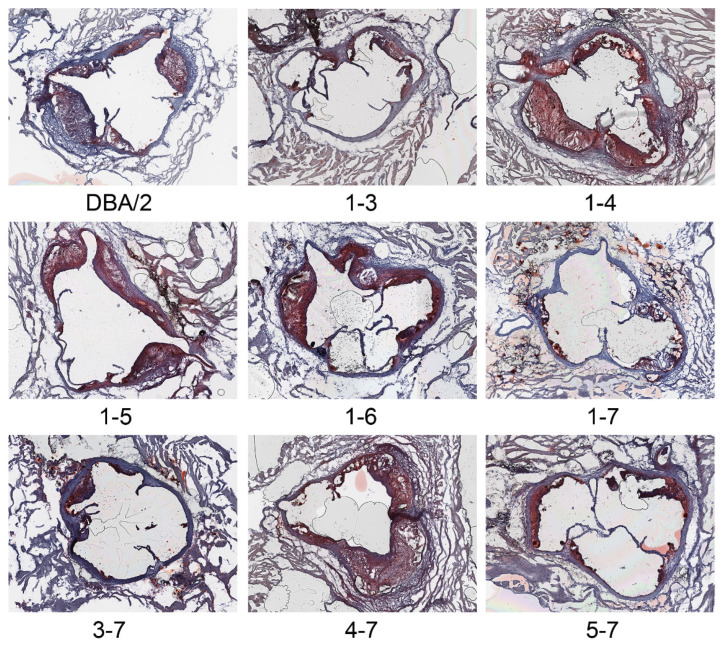
Representative aortic root lesions in female mice from the parental DBA/2 strain and the eight congenic lines stained with Oil red O and hematoxylin. 4× objective lens.

We used marker selection back crossing to create the chr2 *Ath28* locus 1-7 *Apoe*^−/−^ congenic strain, which is on the DBA/2J background with AKR/J alleles only present on chr2 from 171.55 Mb to the last polymorphic marker on the end of the chromosome genotyped on the MegaMuga array (Figure 1, Supplementary Table S2). Areas of uncertainty between genotyped markers or at the end of chr2 are shown in grey lines in Figure 1. Our finding of very few mapped SNPs in these gray regions suggests that they are identical by descent in the AKR/J and DBA/2J genomes, and thus not likely to harbor atherosclerosis modifier genes. After breeding the *Ath28* locus 1-7 line to homozygosity (AA genotype), we found that aortic root lesions were ~50% smaller in both male and female mice, and in combined sex analysis adjusted for the sex effect, compared to the recipient DBA/2J *Apoe*^−/−^ strain (Figure 2A–C, *p* < 0.001). Representative aortic root cross sections for all of the lines are shown in Figure 3. Our data confirmed the presence of an atherosclerosis modifier gene in this interval, and the direction of the effect matched that of the parental strains with DBA/2J *Apoe*^−/−^ having larger lesions than AKR *Apoe*^−/−^ mice [9].

There was no significant difference between line 1-7 and DBA/2J mice in regard to body weight, total, or HDL cholesterol (Table 1). In order to fine map the *Ath28* QTL, we continued backcrossing line 1-7 heterozygotes to DBA/2J *Apoe*^−/−^ mice to generate recombinants that were mapped and bred to homozygosity, yielding seven additional lines: 1-3, 1-4, 1-5, 1-6, 3-7, 4-7, and 5-7 (Figure 1, Supplementary Table S2). Aortic root lesions were reproducibly and significantly smaller in lines 1-3, 3-7, and 5-7 in both males and females, and after combined sex analysis, while the other lines, 1-4, 1-5, 1-6, and 4-7, had lesions similar to the DBA/2J parental line (Figure 2A–C). Our interpretation of this data is that there are three atherosclerosis regulating regions within *Ath28*, called *Ath28.1*, *Ath28.2*, and *Ath28.3* whose positions, including regions of uncertainty between genotyped markers, are shown in Figure 1 and Supplementary Table S3. *Ath28.1* is by far the smallest region, containing only 217 Kb, while *Ath28.2* and *Ath28.3* contain large areas of uncertainty bringing them to 2.7 and 1.8 Mb, respectively (Supplementary Table S3). Compared to DBA/2J alleles, *Ath28.1* and *Ath28.3* appear to harbor atherosclerosis-protective AKR/J alleles, and we assigned them a score of −1 (for direction on lesion area); while *Ath28.2* appears to harbor an atherosclerosis-promoting AKR/J allele, and we assigned it a score of +1. For all eight congenic lines, we summed the scores depending upon the presence or absence of the *Ath28.1*, *Ath28.2*, and *Ath28.3* regions (excluding regions of uncertainty), yielding scores of either 0 (neutral vs. DBA/2J) or −1 (one additional atherosclerosis-protective allele vs. DBA/2J). All of the lines with scores of −1 had significantly smaller lesions vs. DBA/2J reproducibly in males and females, and after adjusting for and combining the sexes (Figure 2A–C). The only other phenotype that replicated in both males and females was elevated HDL cholesterol in line 1-6 (Table 1), which was not one of the lines with smaller lesions. This HDL effect could be due to the presence of two HDL modifier genes with opposing effects, an HDL lowering gene mapping distal to line 1.6, and an HDL raising gene mapping between the distal endpoints of lines 1-5 and 1-6.

We performed a bioinformatics analysis of the strain genetic differences within *Ath28.1*, the smallest region, which overlaps with four long noncoding RNA genes (*Ctcflos*, *Gm36691*, *Gm46780*, and *Pmepa1os*) and three protein coding genes (*Pck1*, *Zpb1*, and *Pmepa1*). The Sanger mouse sequence project identified 531 SNPs that differ between the AKR/J and DBA2/J strains within *Ath28.1* (Supplementary Table S4). The DBA/2J allele matched the C57BL/6 allele for 483 of the 531 SNPs in this region. Most of the SNPs are intergenic or intronic, with one 3’ UTR SNP in the *Zbp1* gene, one 5’ UTR SNP in the *Pmepa1* gene, and one missense SNP in the *Zbp1* gene that changes amino acid residue 280 from a threonine to a serine (AKR/J to DBA/2J). This is a somewhat conservative amino acid change, with a Provean analysis score of −1.245, predicted as a neutral substitution, with a threshold of −2.5 required to predict a detrimental substitution. Another group of 16 SNPs came up as either being downstream of *Pmepa1* or 3’ UTR variants (*n* = 15), or a synonymous variant (*n* = 1). The Sanger mouse sequence project also detected 160 short indels that differ between AKR/J and DBA/2J, with 135 DBA/2J alleles the same as the C57BL/6J reference (Supplementary Table S5). Although most indels were intergenic or intronic, there was also one each in *Zbp1* 5’ and 3’ UTRs, one in the 3’ UTR or downstream of *Pck1*, and six in the 3’ UTR or downstream of *Pmepa1*. There were also two structural variants noted (Supplementary Table S6), one is a 12 bp insertion in AKR/J ~ 4 kb proximal on the chromosome but 3’ of the *Zbp1* gene, and the other is a 439 bp deletion in AKR/J ~600 bp proximal on the chromosome but 3’ of the *Zpb1* gene.

Overlap of the three protein coding genes (and protein names) with atherosclerosis in PubMed yielded 1 hit for *Zbp1*, 9 hits for *Pck1*, and 0 hits for *Pmepa1*. We determined the tissue expression distribution of the human orthologues of these genes. *ZBP1* is expressed highest in spleen (18 transcripts per million (TPM)), whole blood, small intestine, and lung, with low expression in arteries (<1 TPM). *PCK1* is expressed highest in liver (530 TPM), kidney, adipose tissue, pituitary, and small intestine, with some expression in coronary and tibial arteries (>1 TPM). *PMEPA1* is expressed highest in aorta (115 TPM), cervix, tibial artery, prostate, and coronary artery. Searching the human GWAS catalog identified a variant between the *PMEPA1* and *ZBP1* genes associated with herpes zoster infection, and 24 variants in or near the *PCK1* gene associated with red blood cell traits, glomerular filtration rate, parameters of body fat distribution, serum alkaline phosphatase activity, and triglyceride levels.

## 4. Discussion

Although monogenic syndromes in humans that raise LDL-C levels strongly promote coronary artery disease, common variation captured via human GWAS and polygenic risk scores are associated with the vast majority of coronary artery disease events [10]. Thus, the polygenic nature of atherosclerosis has been established in humans, but what about in inbred mouse strains? Here, we took advantage of classical mouse QTL mapping to identify the genetic loci associated with divergent aortic root lesion areas in two inbred mouse strains on *Apoe*^−/−^ background. Considering the fairly large coefficient of variation for aortic root lesions in inbred *Apoe*^−/−^ mice [5], the QTL method will most likely yield only the strongest loci associated with this trait; furthermore, we previously identified three replicated *Ath* QTLs on chr2, 15, and 17 [6]. However, due to the polygenic nature of atherosclerosis, we suspect that even within an *Ath* locus, subcongenic mice might distinguish several distinct modifier loci. Thus, it was not surprising that our subdivision of the *Ath28* QTL via eight congenic strains yielded three distinct regions associated with lesion size. A similar finding was made for another mouse complex trait, diet-induced obesity, where five subcongenic strains for the *Obrq2* (obesity resistance QTL-2) locus on chr6 identified four distinct loci that were associated with this trait [11]. Our confidence for the *Ath28.1*, *Ath28.2*, and *Ath28.3* QTLs harboring three distinct atherosclerosis modifier genes is bolstered by their independent replication in male and female mice.

We are starting our atherosclerosis modifier gene discovery efforts at the *Ath28.1* QTL, as it is the smallest locus and harbors the fewest number of genes, including only three protein coding genes *Pck1*, *Zpb1*, and *Pmepa1*. Depending on what criteria are used, any of these protein coding genes could be the causal modifier gene. *Zbp1* encodes Z-DNA binding protein 1, also known as DAI (DNA-dependent activator of interferon-regulatory factors). It is a prime atherosclerosis modifier gene candidate based on having a missense substitution that might alter protein function, and 3’ UTR SNP and 5’ and 3’ UTR indels that could regulate its mRNA stability or translation. Its high expression in human spleen and whole blood could also be relevant to atherosclerosis. Although *Zbp1* has not yet been studied as an atherosclerosis modifier, it has a role as a sensor of viral infections, in NLRP3 regulation after viral infection, and a role in necroptosis and inflammation via its effects on the RIPK1, RIPK3, and MLKL pathways [11]. The RIPK1-MLKL pathway has repeatedly been shown to play a role in atherosclerosis [12], including RIPK1 studies showing its role in macrophage necroptosis in mouse atherosclerosis, its high expression in early human lesions, and that its knockdown leads to reduced atherosclerotic lesions and inflammatory cytokines in mice [13,14]. This pathway ties in with both the innate and acquired immune systems as well-established modifiers of atherosclerosis initiation and progression [15], as well as the human findings that anti-IL-1b treatment decreased cardiovascular events in the CANTOS trial [16].

*Pck1* encodes the cytosolic phosphoenolpyruvate carboxykinase 1, also known as PEPCK-C, while the unlinked gene *Pck2* encodes the mitochondrial PEPCK-M. PEPCK is a gluconeogenic enzyme, and its expression is under complex hormonal control [17]. We noted one indel that differs in our two mouse strains in the 3’ UTR of *Pck1*, which might alter its mRNA stability. Although *Pck1* expression is important in the liver to mediate gluconeogenesis, its expression is also detected at low levels in human arteries. In addition to the many human GWAS hits for *PCK1* noted above, human studies also identified a SNP in *PCK1* that interacts with the plasma level of omega-3 polyunsaturated fatty acids in modulating insulin resistance in metabolic syndrome subjects [18]. The direct role of *Pck1* in atherosclerosis has not been studied.

*Pmepa1* encodes the prostate transmembrane protein, androgen induced 1; however, this name may be misleading, as the gene is expressed higher in the aorta and tibial artery than in the prostate. This expression pattern combined with many 3’ UTR or downstream SNPs and indels that differ between AKR/J and DBA/2J mice, which may alter its expression, also make this gene a potential atherosclerosis modifier gene. *Pmepa1* has been mostly studied in cancer, where it promotes proliferation, invasion, and migration via a PTEN-AKT pathway [19]. *Pmepa1* also has roles in osteoclast formation and proton production via regulation of vesicular trafficking [20]. To date, *Pmepa1* has not been studied in vascular biology or atherosclerosis.

Each of these protein coding genes can be tested in the future as a mouse atherosclerosis modifier gene, through the use of under- and over-expression models, as well as gene editing to convert the DBA/2J Zbp1 protein into the AKR/J isoform. The significance of our findings includes the confirmation of the polygenic nature of atherosclerosis in a mouse model, the results were internally replicated in both sexes, and in fine mapping the *Ath28.1* QTL to a 217 kb region that contains only three protein coding genes. Thus, this study sets the stage for future gene expression and functional studies in the congenic lines and candidate gene testing studies. These future studies may identify new genes and pathways that play a role in atherosclerosis initiation and/or progression, and thus may provide targets for existing or novel therapeutics.

Mouse models are the most commonly used preclinical models of atherosclerosis. Their relevance to human atherosclerosis is underscored by this disease being driven by high levels of non-HDL cholesterol in both species [1]. In addition, there is strong concordance for many atherosclerosis modifier genes in both species; of 46 genes with human atherosclerosis GWAS signals studied in mouse models, 45 exhibited consistent effects on atherosclerosis or related phenotypes [21]. However, mice are not humans, and it is possible that not all atherosclerosis modifiers will be shared.

One limitation of our study is that due to the large number of strains and mice, atherosclerosis analysis was limited to the lesion areas in the aortic root. Lesion cellular distribution and lesions at other locations will be addressed after atherosclerosis modifier gene identification.

## 5. Conclusions

Atherosclerosis is a complex trait driven my hypercholesterolemia, but subject to many modifiers, including but not limited to those that alter inflammation, and responses of macrophages, endothelial cells, and smooth muscle cells. Taking advantage of classical mouse genetics using *Apoe*^−/−^ mice on the AKR/J and DBA/2J strains, we identified three atherosclerosis-modifying sub-regions within the *Ath28* QTL on the distal end of chr2. Bioinformatic analyses identified all genes and strain differences for the smallest *Ath28.1* QTL sub-region. Additional candidate gene testing studies are required to identify the atherosclerosis modifier genes at each of these sub-regions.

## Figures and Tables

**Table 1:** Body weights, total plasma, and HDL cholesterol levels in DBA/2 and congenic mice.

Mouse Line	Body Wt g Mean ± SD (*n*)	*p*-Value ^a^	Total Cholesterol mg/dL Median [IQR] (*n*)	*p*-Value ^b^	HDL Cholesterol mg/dL Median [IQR] (*n*)	*p*-Value ^b^
			**Males**			
DBA/2	26.2 ± 2.5 (39)		1099 (888–1206) (36)		27 (16–50.2) (27)	
1-7	26.3 ± 2.9 (28)	ns	1089 (951–1217) (28)	ns	43.2 (30.2–62) (22)	ns
1-6	27.0 ± 1.5 (11)	ns	1173 (1016–1345) (11)	ns	54.7 (38–89.1) (8)	*
1-5	24.2 ± 3.5 (7)	ns	1056 (883–1164) (7)	ns	43.6 (36.5–68) (7)	ns
1-4	26.9 ± 2.0 (15)	ns	1077 (870–1120) (15)	ns	38.7 (29.5–50.3) (14)	ns
1-3	26.6 ± 2.1 (24)	ns	1031 (891–1151) (24)	ns	51 (38.4–60.4) (24)	*
3-7	27.2 ± 2.0 (12)	ns	1077 (1000–1131) (12)	ns	30.5 (26.1–43.1) (10)	ns
4-7	24.1 ± 2.2 (22)	**	1111 (1003–1244) (18)	ns	37.2 (26.6–56.6) (16)	ns
5-7	24.7 ± 1.7 (14)	ns	1016 (934–1042) (15)	ns	44.8 (26.6–63.3) (14)	ns
			**Females**			
DBA/2	21.2 ± 2.2 (25)		1123 (935–1245) (25)		29.3 (12.6–39.6) (23)	
1-7	22.2 ±1.8 (28)	ns	1222 (962–1447) (28)	ns	40.2 (24.2–63.1) (21)	ns
1-6	23.8 ±2.3 (5)	ns	1242 (1187–1270) (5)	ns	73.3 (55.7–75.3) (5)	*
1-5	22.5 ±1.9 (7)	ns	1151 (813–1170) (7)	ns	47.5 (39.7–65.8) (7)	ns
1-4	20.5 ±2.0 (8)	ns	890 (797–933) (8)	*	29.2 (18.8–44.6) (8)	ns
1-3	22.4 ±3.0 (16)	ns	951 (839–1086) (16)	ns	21.7 (14.7–57.7) (15)	ns
3-7	23.3 ±1.8 (15)	ns	1230 (1064–1501) (15)	ns	23.5 (17.1–48.3) (15)	ns
4-7	21.4 ±1.9 (19)	ns	1147 (1061–1235) (17)	ns	37.2 (26.6–56.6) (16)	ns
5-7	21.2 ±1.7 (12)	ns	956 (893–1116) (12)	ns	33.2 (23–52.8) (12)	ns

^a^, ANOVA vs. DBA/2J with Dunnett’s multiple comparison test; ^b^, Kruskal–Wallis non-parametric ANOVA vs. DBA/2J with Dunn’s multiple comparison test; IQR, interquartile range; ns, not significant; *, *p* < 0.05; **, *p* < 0.01.

## Data Availability

Supplementary Table S7 provides the body weights, total cholesterol, HDL cholesterol, and atherosclerosis lesion areas values for each mouse of each genotype and sex.

## Supplementary Materials

The following are available online at https://www.mdpi.com/article/10.3390/genes13010070/s1, Table S1: Chromosome 2 SNP markers used to initially identify recombinants (SNPs 1 through 7) and for fine mapping the 3’ end of line 1-4, Table S2: Positions of congenic lines on chr2 including regions of uncertainty between AKR and DBA/2 genotyped markers, Table S3: Positions of the Ath28 fine mapped QTLs including the regions of uncertainty between two mapped markers or at the end of chr2, Table S4: SNPs that differ between AKR/J and DBA/2J mice in the Ath28.1 interval, Table S5: Indels between the AKR/J and DBA/2J strains, Table S6: Structural variants between AKR/J and DBA/2J strains, Table S7: Body weights, total cholesterol, HDL cholesterol, and atherosclerosis lesion areas values for each mouse of each genotype and sex.
