# Supplementary material for: Fine Mapping of the Mouse Ath28 Locus Yields Three Atherosclerosis Modifying Sub-Regions
Source: Genes (Basel). 2021 Dec 28;13(1):70. doi: 10.3390/genes13010070 (PMC8774523; doi:10.3390/genes13010070)
Supplement: Supplementary file 1 [file genes-13-00070-s001.zip › Supplementary Tables S1-3.pdf]

Supplemental Table S1. Chromosome 2 SNP markers used to initially identify recombinants (SNPs 1 through 7) and for fine mapping the 3' end of line 1-4.

SNP Marker #	rs ID	Chr 2 bp (mm9)
1	rs27614159	171550518
2	rs3679486	171571210
3	rs13476925	173100193
4	rs33413718	174686536
5	rs27684224	178358233
6	rs13476938	179308861
7	rs27641595	180092501
1-4.1	rs27606433	174689739
1-4.2	rs27606429	174690998
1-4.3	rs27606278	174728948
1-4.4	rs27650105	177049429
1-4.5	rs27650096	177102650
1-4.6	rs27650082	177276154
1-4.7	rs27650081	177285277
1-4.8	rs27668323	178009095
1-4.9	rs27685256	178133457
1-4.10	rs27685120	178176885

Supplemental Table S2. Positions of congenic lines on chr2 including regions of uncertainty between AKR and DBA/2 genotyped markers.

Line	mm9 bp preceding DBA	mm9 bp 1st AKR	5' uncertain length	mm9 bp last AKR	mm9 bp next DBA	3' uncertain length
5.7	174,689,739	178,349,647	3,659,908	180,092,501	Chr2 end	1,207,499
4.7	173,139,397	173,171,972	32,575	180,092,501	Chr2 end	1,207,499
3.7	172,954,530	173,017,937	63,407	180,092,501	Chr2 end	1,207,499
1.7	166,179,791	171,550,518	5,370,727	180,092,501	Chr2 end	1,207,499
1.6	166,179,791	171,550,518	5,370,727	179,526,213	179,556,799	30,586
1.5	166,179,791	171,550,518	5,370,727	178,567,911	178,830,101	262,190
1.4	166,179,791	171,550,518	5,370,727	174,728,948	177,049,429	2,320,481
1.3	166,179,791	171,550,518	5,370,727	174,301,374	174,312,679	11,305

Supplemental Table S3. Positions of the Ath28 fine mapped QTLs including the regions of uncertainty between two mapped markers or at the end of chr2.

Ath QTL	mm9 bp beginning	mm9 bp end	bp distance
Ath28.1	172,954,530	173,171,972	217,442
Ath28.2	174,301,374	177,049,429	2,748,055
Ath28.3	179,526,213	181,300,000	1,773,787
